# Use of Stereotactic Radioablation Therapy as a Bailout Therapy for Refractory Ventricular Tachycardia in a Patient with a No-entry Left Ventricle

**DOI:** 10.19102/icrm.2021.120902

**Published:** 2021-09-15

**Authors:** Dursun Aras, Huseyin Furkan Ozturk, Elif Ozdemir, Umit Kervan, Meryem Kara, Serkan Cay, Nazim Coskun, Firat Ozcan, Ahmet Korkmaz, Ozcan Ozeke, Serkan Topaloglu, Yilmaz Tezcan

**Affiliations:** ^1^Department of Cardiology, University of Health Sciences, Ankara City Hospital, Ankara, Turkey; ^2^Department of Radiation Oncology, University of Health Sciences, Ankara City Hospital, Ankara, Turkey; ^3^Department of Nuclear Medicine, University of Health Sciences, Ankara City Hospital, Ankara, Turkey; ^4^Department of Cardiovascular Surgery, University of Health Sciences, Ankara City Hospital, Ankara, Turkey

**Keywords:** Mechanical aortic and mitral valve, no-entry left ventricle, refractory ventricular tachycardia, SBRT, stereotactic radioablation therapy

## Abstract

In patients with mechanical aortic and mitral valves and left ventricular (LV) tachycardia (VT), catheter ablation is technically challenging due to the limited access to the LV. Promising new alternatives to radiofrequency ablation include pulsed-field electroporation, percutaneous or surgical sympathetic neuromodulation, and noninvasive stereotactic radioablation therapy (SBRT). We herein describe the effect of SBRT as a bailout therapy on the management of a challenging VT case in the presence of double left-sided mechanical valves.

## Introduction

In patients with mechanical aortic and mitral valves and left ventricular (LV) tachycardia (VT), catheter ablation is technically challenging due to the limited access to the LV. Several operators have reported on the application of non-conventional techniques for VT ablation, including transventricular septal puncture, transmechanical valve, transcoronary venous, and transapical approaches.^1–5^ Promising alternatives to radiofrequency ablation include percutaneous or surgical sympathetic neuromodulation, alcohol ablation from the coronary arterial or venous system, direct current or pulsed-field electroporation, and noninvasive stereotactic radioablation therapy (SBRT) in critically ill patients.^6–9^ We herein describe the effect of SBRT as a bailout therapy on the management of a challenging VT case with two mechanical valve prostheses in aortic and mitral positions.^2^

## Case presentation

**Figure 1:**
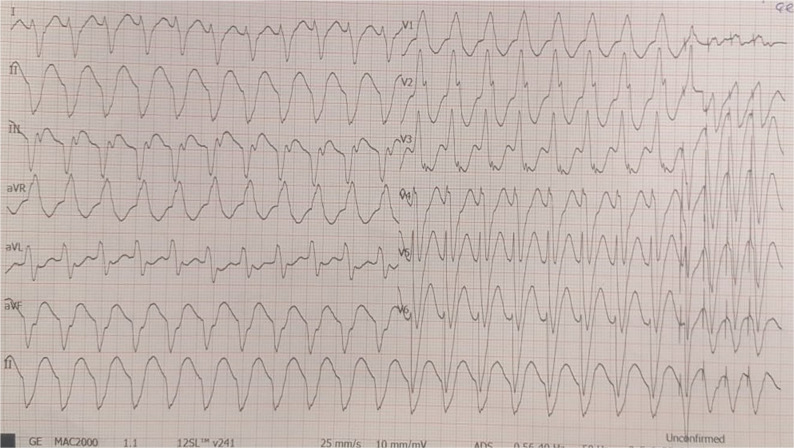
Twelve-lead electrocardiogram showing the patient’s clinical VT suggesting the exit of tachycardia at the left mid-ventricular inferolateral area.

**Figure 2:**
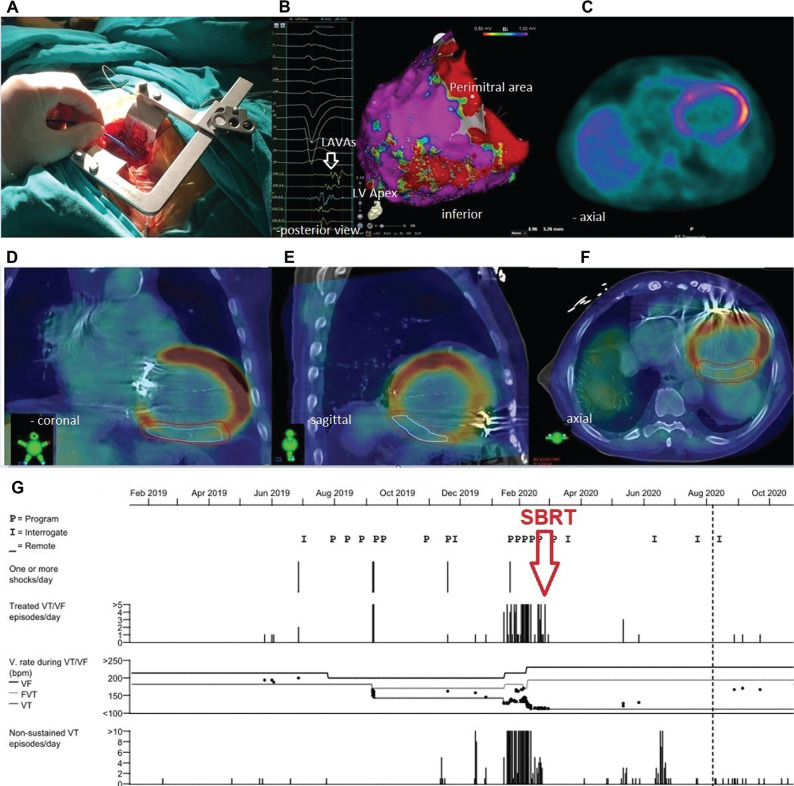
After a mini-thoracotomy **(A)**, three-dimensional mapping **(B)** with images of PET-CT **(C)** and 4D CT **(D–F)** were used to detect the target substrate. The noninvasive SBRT (red arrow) was applied successfully in March 2020 **(G)**. FVT, fascicular ventricular tachycardia; LAVAs, local abnormal ventricular activities; LV, left ventricular; SBRT, stereotactic radioablation therapy; VF, ventricular fibrillation; VT, ventricular tachycardia.

**Figure 3:**
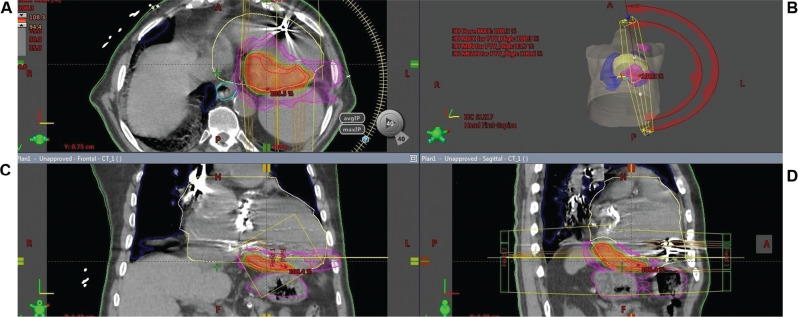
Treatment plan by the isodose distribution of SBRT in axial **(A)**, coronal **(C)**, and sagittal **(D)** orientation is shown with PTV **(B)**. Color lines delineate areas with the same dose (isodose lines). CT, computed tomography; PTV, planned target volume; SBRT, stereotactic radioablation therapy.

A 58-year-old man with two mechanical valves in mitral and aortic positions was referred after multiple appropriate implantable cardioverter-defibrillator shocks despite oral amiodarone and mexiletine therapies. He had nonischemic, valvular, dilated cardiomyopathy with an LV ejection fraction of 15% and had undergone implantation of a cardiac resynchronization therapy (CRT) device 10 years prior. His documented monomorphic VT suggested a lateral mid-LV exit **(Figure 1)**. Due to his previous double valve surgery, we planned and performed an epicardial VT ablation via a lateral thoracotomy **(Figure 2A)**. Electroanatomic mappings (CARTO^®^ 3; Biosense Webster, Diamond Bar, CA, USA) showed scar regions containing local abnormal ventricular activities (LAVAs) in the inferolateral wall of the LV **(Figure 2B)**. Despite epicardial LAVA ablation, after five months, recurrent VT episodes with similar exit sites from previously delineated scar regions recurred under amiodarone, mexiletine, and sotalol therapies. We also transiently switched off the CRT device to exclude the possible lead-related VT, as there are some controversial data concerning the proarrhythmic or antiarrhythmic potential of CRT therapy.^10^ As all available cardiologic therapeutic strategies failed to free the patient from VT recurrence, we decided to implement SBRT as a therapy of last resort after discussions in our group and with the patient and his relatives. In the next step, positron-emission tomography-computed tomography (PET-CT) showed hypometabolic scar areas in the inferolateral walls corresponding with previous electroanatomic maps **(Figure 2C)**. A four-dimensional (4D) CT scan (General Electric Healthcare, Milwaukee, WI, USA) with 10 respiratory phases was performed for radiotherapy planning using an alpha cradle and a chest board for immobilization after an eight-hour fast. After transferring the CT images to the radiotherapy planning system (Eclipse™; Varian Medical Systems, Palo Alto, CA, USA), an average-phase CT (avgCT) was created to evaluate cardiac and respiratory movements. After the fusion of PET-CT and avgCT images using the RT planning software (Varian Medical Systems) **(Figures 2C–2F)**, an inferolateral hypometabolic scar region with the guidance of electroanatomic mapping was created as the internal gross target volume (IGTV). To overcome any uncertainties during positioning, motion, and therapy, a 5-mm safety margin was added to the IGTV for obtaining the final planned target volume (PTV) **(Figure 3)**. Before treatment delivery, re-imaging with cone-beam CT was performed for comparison and alignment of images with simulation images. All treatment procedures were handled by a team of electrophysiologists, radiation oncologists, nuclear medicine specialists, and radiation physicists. Radiotherapy was given using a linear accelerator device (TrueBeam^®^; Varian Medical). A total dose of 25 Gy was delivered in a single fraction to the PTV in 7.5 minutes to be targeted by at least 95% of the given dose. Despite rare hemodynamically stable slow VT episodes responding to antitachycardia pacing, a significant reduction in VT episodes was observed within the first eight months after radioablation therapy **(Figure 2G)**. No abnormal device parameters nor acute or chronic adverse effects were detected at 10 months. At present, the patient is being followed up with at the outpatient clinic.

## Discussion

The growing burden of ventricular arrhythmias and their significant morbidity and mortality rates demand new effective therapeutic approaches based on a given patient’s triggers, comorbidities, and underlying pathology. Mechanical prosthetic aortic and mitral valves impede either a retrograde aortic or transseptal approach to the LV endocardium. SBRT, delivered in a single dose of 25 Gy, may serve as an alternative or palliative therapy for otherwise untreatable patients with refractory VT and electrical storm who are otherwise ineligible for catheter ablation,^6,11–13^ potentially keeping ventricular arrhythmias quiescent while awaiting heart transplantation,^14^ as was done in the current case. Because of its high precision and target conformity, it can deliver a high dose of radiation to a specific area in tissue without significantly affecting the nearby tissues.^15–18^ Indeed, the radiobiology of this treatment when applied to noncancer myocytes is limited, but endothelial vacuolization is a well-described effect of radiation.^19^ There have been studies that have investigated single-fraction whole-heart irradiation in animal models, and these demonstrated dose-dependent myocardial degeneration and fibrosis progressing from the epicardial tissue to full transmurality in the months after irradiation at doses of 20 Gy and higher.^20,21^ Histopathologic findings in the human myocardium after SBRT for recalcitrant VT have also provided radiobiological mechanisms of acute cellular injury during SBRT for VT, which may have an antiarrhythmic effect before the onset of fibrosis.^22^ These data suggest that SBRT can also be used as a relatively acute antiarrhythmic palliation in patients with electrical storm.^21^ Nonetheless, the optimal dose regimen has yet to be elucidated, and the long-term efficacy and toxicity of SBRT remain unsettled. Before more data on efficacy and safety are obtained, it could be offered as a “bailout” procedure after failed endo- and/or epicardial catheter ablation.^12,23^ The current case suggested that the noninvasive SBRT was applicable as a potential salvage treatment option for patients with double mechanical valves with dosage at 25 Gy providing the safest short-term profile. A recent systematic literature review summarized that VT was targeted with reduction in the number of episodes beyond 85% during follow-up, with an encouraging short-term safety profile.^24^ Nevertheless, the same analysis revealed significant heterogeneity in the available study designs, limiting the clinical evidence on the efficacy and safety of SBRT.^24^ Moving forward, randomized clinical trials will be essential to establish the safety, efficacy, and comparative effectiveness of this new and promising modality.^23^
